# Evaluation of the anti-diarrheal activity of the aqueous stem extract of *Lantana camara* Linn (Verbenaceae) in mice

**DOI:** 10.1186/s12906-017-1696-1

**Published:** 2017-04-04

**Authors:** Edlam Tadesse, Ephrem Engidawork, Teshome Nedi, Getnet Mengistu

**Affiliations:** 1 Department of Pharmacology and Clinical Pharmacy, School of Pharmacy, Addis Ababa University, P. O, Box 1176, Addis Ababa, Ethiopia; 2 Department of Pharmacology and Clinical Pharmacy, School of Pharmacy, College of Health and Medical Sciences, Haramaya University, P. O, Box 235, Harar, Ethiopia

**Keywords:** Antidiarrheal activity, Castor oil induced diarrhoea, Anti-enteropooling, Gastrointestinal motility, *L. camara*

## Abstract

**Background:**

Diarrheal disease remains a public health problem in developing countries, including Ethiopia. In order to alleviate this disease, Ethiopian traditional healers use a wide range of medicinal plants from which *Lantana camara* is one of them. The stem of this plant is traditionally used for the treatment of diarrhoea. In addition, this plant is scientifically evaluated to have an antispasmodic effect on in vitro study. The aim of this study was to evaluate the antidiarrheal activity of the aqueous stem extract of *L. camara* Linn in mice.

**Methods:**

The antidiarrheal activity of the extract was investigated using castor oil induced diarrhoea, enteropooling and small intestine transit models. The test groups received various doses (100, 200, and 400 mg/kg) of the extract, whereas positive controls received Loperamide (3 mg/kg) and negative controls received distilled water (10 ml/kg).

**Results:**

In castor oil induced diarrhoea model, the extract, at all test doses, significantly (*p* < 0.001) prolonged diarrhoea onset, decreased the frequency of defecation, and weight of faeces. Similarly, the extract produced a significant (*p* < 0.001) decline in the weight and volume of intestinal contents at all tested doses. In addition, a significant (*P* < 0.001) reduction in the gastrointestinal motility in charcoal meal test was also observed in all doses of the extract. Phytochemical screening of the extract revealed the presence of flavonoids, alkaloids, tannins, and phytosterols that may play a key role in its antidiarrheal activity.

**Conclusion:**

The obtained results of the present study confirm antidiarrheal activity of the stem of *L. camara*, thus provide the scientific basis for the traditional uses of this plant as a treatment for diarrhoea.

## Background

Diarrhoea remains the second leading cause of mortality among children under five years of age next to respiratory infections and kills more young children than AIDS, malaria, and measles combined [1, 2]. This diseases predominantly affect developing countries and of all child deaths from diarrhoea, 78% occur in the African and South-East Asian regions. Ethiopia’s pneumonia and diarrhoea mortality rate are the 5^th^ highest in the world next to India, Nigeria, Pakistan and the Democratic Republic of Congo [3]. Diarrhoea is the 2^nd^ leading cause of death across all ages next to lower respiratory infections and the two-week prevalence of diarrhoea among children under five years of age was 13% in Ethiopia [4, 5].

To alleviate this disease traditional healer in Ethiopia uses different medicinal plants including *Calpurnia aurea*, *Croton marcostachyus*; *Echinops kebercho* whose antidiarrheal activity is evaluated scientifically [6–8] and also the stem of *L. camara* which is not evaluated scientifically*. L. camara* is known by several common names such as black sage, cuasquito, angel lip, flowered sage, shrub verbena, white sage and wild sage all over the world [9]. In Ethiopia, it has a vernacular name of michi-charo (Sheko ethnic group) [10], Enaro (Maale ethnic communities) [11] and Yewef kollo (Gedeoffa and Amharic language) [12]. Traditionally different parts of this plant are used for the treatment of skin infection and febrile illness [10]. The stem of *L. camara* has been also used in the treatment of diarrhoea, by macerating in an aqueous solvent, in Wonago Woreda, Southern Nations Nationalities and the Peoples Regional State of Ethiopia, which is not yet evaluated scientifically [12]. However, there is strong possibility of presence of said activity as there are already a few reports of antidiarrheal effects of leaf extracts of *L. camara* [13, 14].

Diarrhoea is usually a symptom of diseases in the intestinal tract which can be caused by a variety of bacterial (*Escherichia coli*, Vibrio cholerae, Shigella species etc.), viral (Rota virus, Norovirus, Cytomegalovirus etc.) and parasitic organisms (protozoa and helminths) [15]. Ethnopharmacological studies revealed that *L. camara* has been proven to have activities against that diarrhoea causing microorganisms including antibacterial [16], antifungal [17] and anthelmintic [18] activities. In addition, it also possess antimycobacterial [19], antioxidant [20], antinociceptive & anti-inflammatory [21], antimalarial [22], antiulcerogenic [23], and anti-leishmaniasis [24] activities. In vivo studies, indicate the leaves of *L.camara* and, its constituents (Lanthadne A) have antimotility [13] and antidiarrheal activity in mice model of diarrhoea [14]. Furthermore in vitro study on excised rat ileum also indicates this plant showed antispasmodic activity by antagonising the actions of acetylcholine [25] and substances which can alleviate spasm of the gastrointestinal muscles have been postulated to have antidiarrheal activity. So, the aim of this study was to evaluate the antidiarrheal activity of aqueous stem extract of *L. camara* (ASLC) in mice.

## Methods

### Plant material

The stem of *L. camara* was collected from Bishoftu about 48 km South-east of Addis Ababa in November 2013. The plant was identified and authenticated by a taxonomist and a voucher specimen number ET001 was deposited at the National Herbarium, College of Natural Sciences and Computation, Addis Ababa University for future reference. After collection, the stems were cut into pieces and washed gently using distilled water to remove dirt and soil. The cleaned stem pieces were dried under shade to prevent the direct effect of sun and finally ground to coarse powder.

### Experimental animals

Healthy Swiss albino mice of either sex, weighing 20–30 g and aged 6–8 weeks were used for the experiment. The mice were obtained from an animals unit of the Ethiopian Public Health Institute and School of Pharmacy, Addis Ababa University. The animals were housed in plastic cages at room temperature and on a 12 h light–dark cycle and acclimatised for one week before the commencement of the experiment. During the entire period of the study, the animals were supplied with standard pellet diet and tap water ad libitum. Animals were fasted for 18 h prior to the experiment during which food but not water was withheld except for enteropooling test. Care and handling of the animals were according to internationally accepted guidelines [26] and were approved by Research and Ethics committee of School of Pharmacy, Addis Ababa University.

### Preparation of plant extract

Cold maceration technique was used for the extraction of plant material and a total of 200 g of the coarse powder was used. During the process, 100 g of the coarse powder was soaked in an Erlenmeyer flask with 1 L of distilled water and then placed on a shaker (Bibby Scientific Limited Stone Staffo Reshire, UK) tuned to 120 rpm with occasional shaking for 72 h at room temperature. The extract was filtered first using a muslin cloth and then Whatman grade No-1 filter paper and the marc was re-macerated for a second and third time by adding another fresh solvent [27]. The filtrates were left overnight in a deep freezer and then lyophilized using freeze dryer. After drying, percentage yield of crude aqueous stem extract of *L. camara* was determined to be 16.7% *w*/w. The dried plant extract was reconstituted with distilled water for oral administration.

### Acute oral toxicity test

The acute oral toxicity study was carried out for ASLC by using the limit test recommendations of OECD 420 Guideline [28]. Five healthy adult female Swiss albino mice weighing between 25 to 30 g were used for the study. First, female Swiss albino mouse was fasted (with free access to water) for 4 h and then loaded with 2000 mg/kg of the extract, orally. The mouse was then fasted for 2 h and strictly observed for the general signs and symptoms of toxicity, food, water intake and mortality within 24 h. Since no death was observed within 24 h, additional four mice were fasted for 4 h and administered the same dose of the extract followed by 2 h fasting. The animals were observed continuously for 4 h with 30 min interval and then for 14 consecutive days with an interval of 24 h for the general signs and symptoms of toxicity, food and water intake and mortality.

### Experimental design

#### Animal grouping and dosing

In all models, animals were randomly divided into five groups (negative control, positive control and three test groups) comprising of six animals in each group. Negative controls received vehicle (10 ml/kg, distilled water) and positive controls received Loperamide (3 mg/kg) in all models. The test groups (group 3,4 and 5) received different doses (100, 200 and 400 mg/kg respectively) of the ASLC orally which were determined based on the acute oral toxicity test and pilot study.

### Determination of anti-diarrheal activity

#### Castor oil induced diarrhoea

The method described by Awouters et al. [29] was followed for this study. Swiss albino mice of either sex were fasted for 18 h with free access to water and grouped and treated as described under grouping and dosing section. One hour after treatment, diarrhoea was induced by oral administration of 0.5 ml castor oil to each mouse. The animals were then housed in a separate transparent cage in which the floor is lined with white paper. The paper was changed every hour for a total of four hours. During the observational period, the onset of diarrhoea, number and weight of wet stools, total number and the total weight of faecal output were recorded. Finally, the percentage of faecal output (% FOP) and diarrheal inhibition (% inhibition of defecation) were calculated by using the formulas described below.

$$ \%\mathrm{of}\ \mathrm{faecal}\ \mathrm{output}=\frac{\mathrm{Mean}\ \mathrm{faecal}\ \mathrm{weight}\ \mathrm{of}\ \mathrm{each}\ \mathrm{treatment}\ \mathrm{group}}{\mathrm{Mean}\ \mathrm{faecal}\ \mathrm{weight}\ \mathrm{of}\ \mathrm{control}\ \mathrm{group}} X\ 100 $$

$$ \%\mathrm{Inhibition}\ \mathrm{of}\ \mathrm{defaecation}=\frac{\mathrm{Mo}{\textstyle -}\mathrm{M}}{\mathrm{Mo}} X\ 100 $$

Where, Mo: Mean defecation of control, M: Mean defecation of test sample/standard drug.

### Castor oil induced enteropooling

The method described by Robert et al. [30] was used to determine the effect of the extract on intraluminal fluid accumulation. For this experiment Swiss albino mice were fasted for 18 h and grouped and treated, as described under grouping and dosing section, 1 h before oral administration of castor oil (0.5 ml/mouse). One hour after castor oil administration, the mice were sacrificed by cervical dislocation. The abdomen of each mouse was open and the whole length of the intestine, from the pylorus to the caecum, was ligated, dissected and carefully removed. The small intestines were weighed and the intestinal contents were collected by milking into a graduated tube to measure the volume. The empty intestines were reweighed and the difference between the two weights was calculated. Finally, the percentage of reduction of intestinal secretion and weight of intestinal contents were determined by using the following formulas.

$$ \%\mathrm{of}\ \mathrm{inhibition}\ \mathbf{by}\ \mathrm{using}\ \mathrm{MVSIC}=\frac{\mathrm{MVICC}-\mathrm{MVICT}}{\mathrm{MVICC}}\times 100 $$

Where MVSIC is the mean volume of the small intestinal content, MVICC is the mean volume of the intestinal content of the control group, & MVICT is the mean volume of the intestinal content of the test groups.

$$ \%\mathrm{of}\ \mathrm{inhibition}\ \mathbf{by}\ \mathrm{using}\ \mathrm{MWSIC}=\frac{\mathrm{MWICC}-\mathrm{MWICT}}{\mathrm{MWICC}}\times 100 $$

Where MWSIC is the mean weight of the small intestinal content, MWICC is the mean weight of the intestinal content of the control, and MWICT is the mean weight of the intestinal content of the test group.

### Gastrointestinal motility test

The effect of ASLC on normal gastrointestinal transit and castor oil-induced intestinal motility was assessed in this test. Mice were fasted for 18 h with free access to water and grouped and treated as described under grouping and dosing section. One hour after treatment each mouse was given 1 ml of 5% charcoal suspension, for the normal gastrointestinal motility test, and 0.5 ml of castor oil for castor oil-induced intestinal motility test. One hour after castor oil administration, all mice were received 1 ml of 5% activated charcoal suspension. The animals were then sacrificed by cervical dislocation after 30 min of administering castor oil and the entire length of the intestine (from the pylorus to the cecum) was removed and placed lengthwise on a white paper. The distance travelled by the charcoal meal and the total length of the intestine was then measured. The peristaltic index and percentage of inhibition were calculated by using the following formula [31].

$$ \mathrm{Peristalsis}\ \mathrm{index}=\frac{\mathrm{Distance}\ \mathrm{travelled}\ \mathrm{by}\ \mathrm{charcoal}\ \mathrm{meal}\ }{\mathrm{Length}\ \mathrm{of}\ \mathrm{small}\ \mathrm{intestine}}\times 100 $$

$$ \kern0.75em \%\mathrm{inhibition}=\frac{\mathrm{Dc}-\mathrm{Dt}}{\mathrm{Dc}}\times 100 $$

Where, Dc: Mean distance travelled by the control, Dt: Mean distance travelled by the test group.

### In vivo antidiarrheal index

In vivo antidiarrheal index (ADI*)* of positive control and extract treated group was determined by using different data from the above tests using the formula developed by Aye-Than et al. [30].

$$ \mathrm{ADI}\  in\  vivo=\sqrt[3]{\ \mathrm{D}\ \mathrm{freq}\times \mathrm{G}\ \mathrm{meq}\times \mathrm{P}\ \mathrm{freq}} $$

Where Dfreq is the delay in defecation time or diarrhoea onset obtained from castor oil diarrheal test by

$$ \mathrm{Drefq}=\frac{\mathrm{mean}\ \mathrm{onset}\ \mathrm{of}\ \mathrm{diarrhoeain}\ \mathrm{the}\ \mathrm{test}\ \mathrm{group}{\textstyle -}\mathrm{mean}\ \mathrm{onset}\ \mathrm{of}\ \mathrm{diarrhoeain}\ \mathrm{the}\ \mathrm{control}\times 100}{\mathrm{mean}\ \mathrm{onset}\ \mathrm{of}\ \mathrm{diarrhoeain}\ \mathrm{the}\ \mathrm{control}\ \mathrm{group}} $$

Gmeq is the gut meal travel reduction (as % of control) obtained from charcoal meal test (% inhibition), and Pfreq is the purging frequency or reduction in the number of wet stools (as % of control) obtained from castor oil diarrheal model (% inhibition of defecation).

### Phytochemical screening

The qualitative phytochemical investigation of ASLC was carried out using standard tests [32, 33] to determine the presence of secondary metabolites such as terpenoids, flavonoids, alkaloids, tannins, saponins, glycosides and phytosterols.

### Statistical analysis

The experimental results were analysed using the Statistical Package for the Social Sciences (SPSS), version 16.0 software. Results are expressed as a mean ± standard error of the mean (SEM), and statistical analyses were carried out by employing one-way analysis of variance (ANOVA), followed by Tukey post Hoc test to compare results with controls and among groups. In all cases, statistical significance was set at *p* < 0.05.

## Results

### Acute oral toxicity test

Oral administration of the ASLC produced neither overt toxic signs nor death during the observation period of 14 days after a single administration of 2000 mg/kg. In addition, neither food nor water intake was found to be reduced during the period. The absence of mortality and signs of overt toxicity up to five times the selected maximum effective dose suggest ASLC have a wider safety margin and LD_50_ value greater than 2000 mg/kg in mice.

### Effect on castor oil- induced diarrhoea

**Fig. 1:**
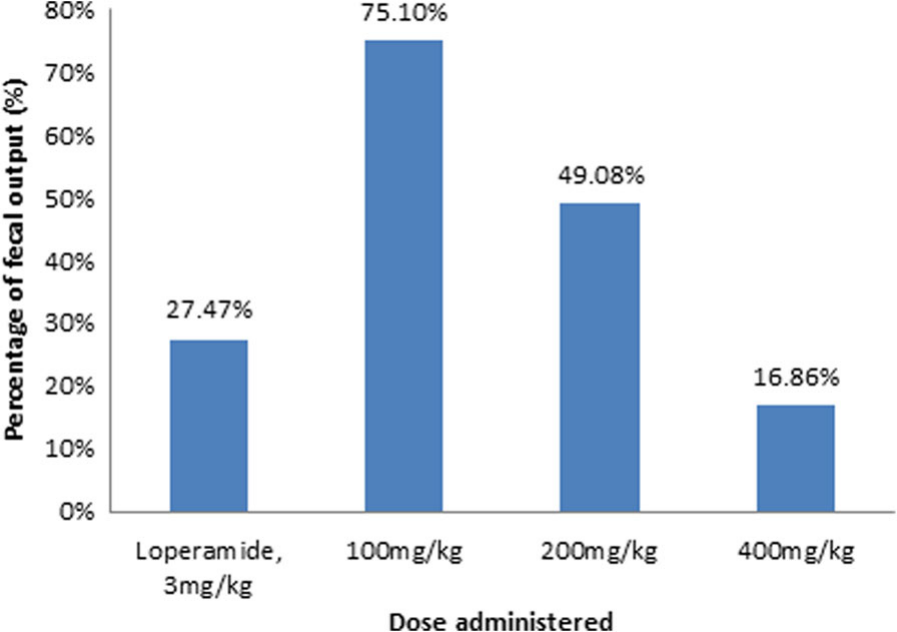
Percentage mean fecal output of the aqueous stem extract of *L. camara* on castor oil induced diarrhea model in mice

Castor oil administration to mice induced diarrhoea for the following 4 h in the control group. This condition was markedly reduced by Loperamide (77.5%) as well as the extract, with the maximum effect observed at 400 mg/kg (87.6%) as shown in Table 1. Oral pretreatment of mice with different doses of the extract showed a significant (*p* < 0.001) delay on the onset of diarrhoea, with the higher dose of the extract exhibiting the better effect. In addition, the extract significantly reduced the frequency of defecation and the number of wet stools when compared with control (*p* < 0.001). Percentage of faecal output was also reduced by different doses of the extract, in which the higher dose of the extract (400 mg/kg) producing a better effect compared to any of the groups as depicted in Fig. 1.

**Table 1:** Effect of the aqueous stem extract of *L. camara* on castor oil induced diarrhea in mice

Treatment	Dose (mg/kg, p.o)	Onset of diarrhea (min)	Total # of feces	Total # of wet feces	Total weight of feces (gm)	Total weight of wet feces (gm)	% Inhibition of defecation
Control	-	13.17 ± 1.08	9.67 ± 0.33	6.67 ± 0.33	0.46 ± 0.01	0.39 ± 0.009	–
Loperamide	3	93.33 ± 2.40^a3c3d3e3^	3.5 ± 0.22^a3c3e1^	1.5 ± 0.22^a3c2^	0.13 ± 0.008^a3c3d3^	0.08 ± 0.007^a3c3^	77.5
ASLC	100	35.67 ± 1.50^a3d3e3^	6.83 ± 0.31^a3d3e3^	3.17 ± 0.31^a3d2e3^	0.34 ± 0.016^a3d3e3^	0.24 ± 0.022^a3d3e3^	52.4
ASLC	200	68.33 ± 2.09^a3e3^	4.50 ± 0.22^a3e3^	1.67 ± 0.21^a3^	0.22 ± 0.015^a3e3^	0.14 ± 0.017^a3e3^	75.0
ASLC	400	185.33 ± 3.72^a3^	2.33 ± 0.21^a3^	0.83 ± 0.17^a3^	0.08 ± 0.007^a3^	0.04 ± 0.008^a3^	87.6

All values are expressed as mean ± standard error of the mean (SEM), (*n* = 6); Data was analyzed by one way ANOVA followed by Tukey post hoc test; ^a^compared to the control, ^b^to the standard drug, ^c^to 100 mg/kg, ^d^to 200 mg/kg, ^e^to 400 mg/kg; ^1^ *p* < 0.05, ^2^ *p* < 0.01, ^3^ *p* < 0.001

*ASLC* aqueous stem extract of *L. camara.* Mice in the control group received distilled water (10 ml/kg)

### Effect on castor oil- induced enteropooling

The volume and weight of intestinal content were significantly (*p* < 0.001, *p* < 0.001) reduced by the extract in a dose-dependent manner (R^2^ = 0.790, R^2^ = 0.814) as compared with control respectively as shown in Table 2. The highest effect on both volume and weight of intestinal content was achieved at 400 mg/kg of the extract.

**Table 2:** Effect of the aqueous stem extract of *L. camara* on castor oil induced intraluminal fluid accumulation in mice

Treatment	Dose (mg/kg, p.o)	MWSIC (gm)	% inhibition by using MWSIC		MVSIC (ml)	% inhibition by using MVSIC	
Control		0.65 ± 0.017	–	R^2^ = 0.790	0.50 ± 0.026	–	R^2^ = 0.814
Loperamide	3	0.16 ± 0.007^a3c3d2e3^	75.32		0.12 ± 0.007^a3c3e2^	76.77	
ASLC	100	0.41 ± 0.013^a3d3e3^	37.53		0.30 ± 0.013^a3d3e3^	38.73	
ASLC	200	0.24 ± 0.013^a3e3^	62.98		0.18 ± 0.019^a3e3^	64.65	
ASLC	400	0.07 ± 0.004^a3^	88.69		0.06 ± 0.003^a3^	88.89	

All values are expressed as mean±standard error of the mean (SEM), (*n* = 6); Data was analyzed by one way ANOVA followed by Tukey post hoc test; ^a^compared to the control, ^b^to the standard drug, ^c^to 100 mg/kg, ^d^to 200 mg/kg, ^e^to400 mg/kg; ^2^ *p* < 0.01, ^3^ *p* < 0.001

*ASLC* aqueous stem extract of *L. camara, MWSIC* mean weight of small intestinal content, *MVSIC* mean volume of small intestinal content. Mice in the control group received distilled water (10 ml/kg), *R* ^*2*^ coefficient of determination

### Effect on gastrointestinal motility

ASLC reduced normal gastro-intestine motility and castor oil induced movement significantly at all doses (*p* < 0.001) when compared with control as shown in Tables 3 & 4 respectively and the maximum effect is obtained by 400 mg/kg on normal gastrointestinal movement (73.62%).

**Table 3:** Effect of the aqueous stem extract of *L. camara* on normal gastrointestinal transit in mice

Treatment	Dose (mg/kg, p.o)	Mean length of small intestinal (cm)	Mean distance traveled by charcoal (cm)	Peristalsis index (%)	% of inhibition
Control		55.87 ± 1.27	38.03 ± 0.26	68.08	–
Loperamide	3	55.43 ± 0.76	10.38 ± 0.36	18.73^a3c3d3^	72.70
ASLC	100	55.33 ± 1.04	25 ± 0.49	45.18^a3d2e3^	34.27
ASLC	200	54.92 ± 1.11	15.52 ± 0.49	28.25^a3e3^	59.20
ASLC	400	56.42 ± 0.81	10.03 ± 0.27	17.78^a3^	73.62

All values are expressed as mean±standard error of the mean (SEM), (*n* = 6); Data was analyzed by one way ANOVA followed by Tukey post- hoc test; ^a^compared to the control, ^b^to the standard drug, ^c^to 100 mg/kg, ^d^to 200 mg/kg, ^e^to 400 mg/kg; ^2^ *p* < 0.01, ^3^ *p* < 0.001

*ASLC* aqueous stem extract of *L. camara*. Mice in the control group received distilled water (10 ml/kg)

**Table 4:** Effect of the aqueous stem extract of *L. camara* on castor oil induced gastrointestinal transit in mice

Treatment	Dose (mg/kg, p.o)	Mean length of small intestinal (cm)	Mean distance traveled by charcoal (cm)	Peristalsis index (%)	% of inhibition
Control	–	55.60 ± 0.85	47.12 ± 0.87	84.74	–
Loperamide	3	55.83 ± 1.30	16.73 ± 0.42	29.97^a3c3d3^	64.49
ASLC	100	57.75 ± 1.02	36.05 ± 0.64	62.42^a3d2e3^	23.49
ASLC	200	57.00 ± 0.84	22.68 ± 0.47	39.80^a3e3^	51.86
ASLC	400	55.12 ± 1.67	15.85 ± 0.41	28.76^a3^	66.36

All values are expressed as mean±standard error of the mean (SEM), (*n* = 6); Data was analyzed by one way ANOVA followed by Tukey post hoc test; ^a^compared to the control (treated with distilled water), ^b^to the standard drug, ^c^to 100 mg/kg, ^d^to 200 mg/kg, ^e^to 400 mg/kg; ^2^ *p* < 0.01, ^3^ *p* < 0.001

*ASLC* aqueous stem extract of *L. camara.* Mice in the control group received distilled water (10 ml/kg)

### In vivo anti-diarrheal index

The result from in vivo ADI revealed that there was a dose-dependent increment in the antidiarrheal index value of ASLC. Moreover, the highest anti-diarrheal index was observed at the maximum dose, 400 mg/kg, of the extract as shown in Table 5.

**Table 5:** In vivo anti-diarrheal index value of the aqueous stem extract of *L. camara*

Treatment	Dose (mg/kg, p.o)	Delay in defecation time (Dfreq)	Gut meal travel reduction (Gmeq)	Purging frequency (Pfreq)	Antidiarrheal index ADI
Control		–	–	–	–
Loperamide	3	608.65	64.49	77.5	144.9
ASLC	100	170.84	23.49	52.4	59.5
ASLC	200	418.83	51.86	75.0	117.7
ASLC	400	1307.21	66.36	87.6	196.6

### Phytochemical screening

Phytochemical screening of the aqueous stem extract of *L. camara* revealed that the presence of flavonoids, alkaloids, tannins, glycosides, phytosterols and saponin as major constituents (Table 6).

**Table 6:** Preliminary phytochemical screening of the aqueous stem extract of *L. camara*

Secondary metabolites	ASLC
Terpenoids	_
Flavonoids	+
Alkaloids	+
Tannins	+
Glycosides	+
Phytosterols	+
Saponin	+

*ASLC* Aqueous stem extract of *L. camara*; +: present; −: absent

## Discussion

The present study was aimed to evaluate the antidiarrheal activity of the aqueous stem extract of *L. camara* by using different experimental models of diarrhoea in mice. In all models, diarrhoea was induced by administering castor oil to each mouse. Castor oil produces diarrhoea due to its active metabolite, a ricinoleic acid which is liberated by the action of lipases in the upper part of the small intestine [34]. It mediates its action by binding to EP3 prostanoid receptors on smooth muscle cells [35] and facilitates the accumulation of fluid in the intestine by inhibiting absorption and enhancing secretion of fluid and electrolytes [36]. Furthermore, this metabolite also alters the motility of GI smooth muscles [37].

In the castor oil induced diarrhoea model, the extract produced a significant effect on all parameters measured: onset of diarrhoea, the number of wet and total stools and weight of wet stools. This result is in concordance with the report on the aqueous leaf extract of *Momordica charantia* [38] and methanol fraction of the leaves of *L. camara* [14]. A previous study suggested that the analgesic and anti-inflammatory activities demonstrated by *L. camara* was due to the inhibition of prostaglandin biosynthesis like that of non-steroidal anti-inflammatory drugs [39]. Thus, the antidiarrheal action exerted by the extract may also be associated with the inhibition of prostaglandin formation. This suggestion is validated by the facts that castor oil induced diarrhoea is related to stimulation of prostaglandins biosynthesis [30, 40].

The phytochemical analysis of the extract revealed the presence of different bioactive agents. Among the secondary metabolite identified flavonoids and phytosterols are known to modify the production of cyclooxygenase 1 and 2 (COX-1, COX-2) and lipooxygenase (LOX) thereby inhibiting prostaglandin production [41, 42]. Tannins present in the extract precipitate the proteins in the intestinal mucosa by forming the protein tannates, which make the intestinal mucosa more resistance to chemical alteration and hence reduce the peristaltic movements and intestinal secretion [43]. Therefore, the anti-diarrheal activity of *L. camara* stem crude extract observed in this study may be attributed to the presence of flavonoids, alkaloids, tannins and phytosterols in the crude extract.

Mostly, antidiarrheal agents act by decreasing secretion and/or reducing the propulsive movement of GI smooth muscles. So to further get information about the mechanism for the antidiarrheal activity, the extract was evaluated by using enteropooling and motility tests.

In the castor oil induced enteropooling assay, the extract significantly reduced the intraluminal fluid accumulation when compared to the negative control. This result was in line with the report of a study by Abel et al. [44] on aqueous extract of *Phoenix dactylifera* and Getnet et al. [14] specifically the aqueous and methanol fractions. The maximal effect of the extract was similar to Loperamide, which is one of the most widely employed drugs against diarrhoea disorder; as shown in present study Loperamide effectively antagonised diarrhoea induced by castor oil [45]. The active metabolite of castor oil, ricinoleic acid, induces irritation and inflammation of the intestinal mucosa, leading to release of prostaglandins. The prostaglandins thus released stimulate secretion by preventing the reabsorption of sodium chloride and water [46]. Thus, it is possible that the extract significantly inhibits gastrointestinal hypersecretion and enteropooling by increasing reabsorption of electrolytes and water or by inhibiting induced intestinal accumulation of fluid.

The anti-enteropooling activity of the extract could also probably be related to the existence of phytochemical constituent including flavonoids, steroids and tannins. Flavonoids and steroids inhibit the release of prostaglandins; thereby inhibit secretion induced by castor oil and facilitate absorption of electrolytes [41, 42]. Tannins decrease fluid secretion by inhibiting CFTR and CaCC, by generating a protein-precipitating reaction to the GI mucosa [43, 47].

In the small intestinal transit test, the extract was able to inhibit intestinal motility; a rising tendency of the inhibitory effect on the gastrointestinal motility was observed when the dose was increased. During the experiment, the charcoal meal method was selected to follow the displacement of the gastrointestinal content because the reduction of gastrointestinal motility is one mechanism by which many antidiarrheal agents can act [48, 49]. The extract significantly reduced intestinal transit as observed by the decrease in GI motility of the charcoal meal. This finding suggests that the extracts act on all parts of the intestine. A decrease in the motility of gut muscles increases the stay of substances in the intestine. This allows a greater time for absorption [50]. Thus, the reduction in the intestinal propulsive movement in the charcoal meal model may be due to the anti-motility property of the extract. This assumption is further supported by the antimotility effect of the leaves of *L.camara* and, its constituents (Lanthadne A) and solvent fractions of the leaves of this plant [13, 14].

Drugs that inhibit intestinal transit in pathophysiological states are effective in relieving diarrhoea [51]. During analysing the result the extract significantly inhibited GI transit in the pathophysiological state as compared with the control. However, the extract was more effective in the normal intestinal transit than castor oil induced intestinal transit. This finding may be due to the constipating activity of the extract at different selected doses. Secondary metabolites such as flavonoids [52] and tannins [43] are reported to possess anti-diarrheal activity due to their ability to inhibit intestinal motility. Hence, the significant anti-motility effect of the extract may be related to the synergistic inhibitory effect of flavonoids and tannins on castor oil induced gastrointestinal motility.

Like the castor oil induced and enteropooling diarrheal model, maximum effect was observed with the highest dose of the extract rather than the standard drug in charcoal meal test. This might be due to different secondary metabolites in the extract that may prolong the time for absorption of water and electrolytes through hampering the peristaltic movement of the intestine.

Clinically, diarrhoea may result from disturbed bowel function, in which case, there is impaired intestinal absorption, excessive intestinal secretion of water and electrolytes and a rapid bowel transit [53]. In vivo*,* ADI is a measure of the combined effects of the different components of diarrhoea, including purging frequency, the onset of diarrheal stools and frequency of intestinal movement [31]. Besides, higher ADI value is a measure of the effectiveness of an extract in curing diarrhoea. The ADI value increased with dose, suggesting the dose dependency of this parameter. The highest selected dose of the extract, with the highest ADI value, is endowed with the best anti-diarrheal activity when compared with other selected doses as indicated on the above results.

## Conclusion

The results of this study revealed that the aqueous stem extract of *L. camara* endowed with significant anti-diarrheal activity. It inhibited the frequency of defecation and reduced greatly the wetness of faecal excretion. Moreover, it also produces an inhibitory effect on castor oil induced intestinal secretion and gastrointestinal propulsion. These antidiarrheal activities of the extract may be attributed to the presence of phytochemicals including tannins, alkaloids, saponins, flavonoids and phytosterols that act individually or collectively. These findings provide a scientific support for a traditional use of the stem of *L. camara* as diarrhoea remedy.

## Abbreviations

ADI: Anti-diarrheal index
ASLC: Aqueous Stem extract of *L. camara*
